# Subsite Distribution of Gastric Cancer in an Area of High Prevalence—Northwest Iran

**DOI:** 10.2188/jea.JE20080044

**Published:** 2009-07-05

**Authors:** Yousef Bafandeh, Sara Farhang

**Affiliations:** 1 Professor of Gastroenterology and Hepatology Liver and Gastrointestinal Diseases Research Center Tabriz University of Medical Sciences, Tabriz, Iran; 2 General practitioner, Liver and Gastrointestinal Diseases Research Center Tabriz University of Medical Sciences, Tabriz, Iran

**Keywords:** gastric cancer, subsite, gastric cardia, Iran

## Abstract

**Background:**

The aim of the present study was to determine subsites of gastric cancer in East Azerbaijan, Iran—a high incidence region for gastric cancer and *Helicobacter pylori* infection.

**Methods:**

Data were collected from 2002 through 2007 from patients who sought treatment for gastrointestinal symptoms or signs at a university clinic and subsequently underwent upper gastrointestinal endoscopy.

**Results:**

Cancer was diagnosed and histologically confirmed in 362 patients (352 adenocarcinomas). The mean age of the patients was 64.57 ± 11.32 (range, 16–94 years) and the male-to-female ratio was 2.8:1. The gastric cardia was involved in 40.3% of patients with gastric adenocarcinoma, while the gastric fundus was involved in 3.7%, the gastric body in 49.1%, and the gastric antrum in 24.1% of patients. Complete evaluation for metastasis was possible in 144 patients; 61 were free of metastasis, and most of these patients underwent surgical therapy. Cardia involvement was not associated with the sex or age of patients.

**Conclusions:**

Noncardia gastric cancer is still more frequent in East Azerbaijan, which is likely due to the very high prevalence of infection with *Helicobacter pylori*. The low rate of cancer involving the fundus is a target for further research on the etiology of gastric cancer.

## INTRODUCTION

Gastric adenocarcinoma is the second leading cause of cancer-related mortality in the world.^1^ Although the incidences of adenocarcinomatous and nonadenocarcinomatous noncardia gastric cancer have decreased, the incidence of adenocarcinoma of the gastric cardia has increased in most developed countries.^2^^–^^5^ Differences in the etiology of stomach cancers at these 2 subsites (cardia vs noncardia) may explain these conflicting trends in incidence. Noncardia gastric cancer is associated with *Helicobacter pylori* (*H pylori*)-induced atrophic gastritis,^6^ while cardia cancer is not associated with colonization of *H pylori* and the level of acid secretion remains normal.^7^

Data on trends in gastric adenocarcinoma are lacking in developing countries. The high ratio of distal versus proximal gastric adenocarcinoma did not change in Turkey during the last decade of the 20th century,^8^ which is due to the fact that *H pylori* infection is the essential causal factor in noncardia gastric adenocarcinoma.^9^ This underscores the role of *H pylori* in the epidemiology of the disease in developing countries.

In Iran, cancer of the stomach is the most common cancer of the gastrointestinal tract and its incidence is high in the region of Azerbaijan, in northwest Iran.^10^^,^^11^ Moreover, the prevalence of infection with *H pylori*, which is known to be involved in the development of gastric adenocarcinoma,^12^^,^^13^ is more than 85% in the adult population of this region.^14^ No data are available on the epidemiology of gastric cancer in this population. Such data would reveal the leading causes and ideal interventions for this common and frequently fatal cancer.

We hypothesized that the characteristics of etiologic factors in this population (including dietary habits and the high prevalence of *H pylori*) result in a subsite distribution of gastric cancer that lies between those observed in Asian and Western countries. We therefore conducted a detailed assessment of gastric cancer tumor subsites in patients from East Azerbaijan.

## METHODS

We studied patients who presented to the referral clinic of Tabriz Medical University and required upper gastrointestinal endoscopy for a chief complaint, or alarming signs or symptoms, related to the gastrointestinal tract. In the period from January 2002 through December 2007, all such patients with endoscopically diagnosed gastric cancer that was subsequently confirmed by histopathology were recruited for the study. The study was approved and supported by the liver and gastrointestinal diseases research center.

Gastroesophageal junction cases were defined as those with cancer originating at the anatomic junction of the esophagus and the stomach. Cardia cases were those with cancer originating within the first 5 cm of gastric mucosa distal to the gastroesophageal junction.^15^ Cancer diagnoses involving more than 1 site were also included in the analysis.

Statistical comparisons were made by the univariate chi-square test or logistic regression for nominal variables and by the independent samples *t*-test or ANOVA for difference of means. The *P* value was set at 0.05 for statistical significance.

## RESULTS

During the period of this cross-sectional study, a diagnosis of cancer was confirmed by histopathology of endoscopic biopsies from 362 patients. The participants included 267 men (73.8%) and 95 women (26.2%). Mean age (SD) of patients at initial diagnosis was 64.9 (11.4) in men and 63.5 (10.9) in women (*P* = 0.319).

Distribution of patients’ complaints were as follows: epigastric pain (58.0%), dysphagia (36.1%), vomiting (26.7%), anorexia (6.3%), upper gastrointestinal tract bleeding (14.9%), weight loss (15.1%), unexplained anemia (2.2%), previous partial gastrectomy for peptic ulcer disease (0.8%), determining origin of a metastasis (0.8%), family history of gastric cancer (0.2%), and hiccups (0.2%).

At the time of initial diagnosis 144 patients were fully evaluated for possible metastasis: 61 (42.3%) had no evidence of metastasis. Surgical resection was possible in 56 (15.5%) patients with gastric cancer, while 11 (3.0%) received chemotherapy and 111 (30.7%) underwent palliative interventions.

Among 362 patients with gastric cancer, 8 had malignant lymphoma, 1 had a gastrointestinal stromal tumor, and 1 had a carcinoid tumor. Age and sex distribution, as well as gastric adenocarcinoma tumor subsite, are shown in the Table. The mean age (SD) of patients at initial diagnosis was 65.1 (11.1) years in men and 63.8 (10.9) years in women (*P* = 0.358).

**Table.** Characteristics of gastric adenocarcinomas in Iranian patients

Site		No (%) of patients	No (%) Female	No (%) Male	Mean (SD) age
Proximal	Gastroesophageal junction and cardia	26 (7.4)	9 (9.9)	17 (6.5)	64.9 (10.5)
	Gastric cardia	67 (19.0)	16 (17.6)	51 (19.5)	64.8 (11.6)
	Gastric fundus	8 (2.3)	1 (1.1)	7 (2.7)	63.5 (9.9)
	Cardia and fundus	1 (0.1)	0	1 (0.4)	65
Middle	Gastric body	121 (34.4)	26 (28.6)	95 (36.4)	64.3 (11.3)
Distal	Gastric antrum	77 (21.9)	25 (27.5)	52 (19.9)	65.6 (12.1)
Diffuse	Cardia and body	44 (12.5)	13 (14.3)	31 (11.9)	66.2 (7.6)
	Body and antrum	4 (1.1)	1 (1.1)	3 (1.1)	62.0 (11.7)
	Linitis plastica (Diffuse)	4 (1.1)	0	4 (1.5)	61.2 (12.2)

	Total	352 (100)	91 (100)	261 (100)	64.86 (10.9)

Cardia involvement (including cancers involving multiple regions) was observed in 39.2% of patients with gastric cancer, and in 40.3% of patients with gastric adenocarcinoma. No significant difference in the distribution by sex was noted: cardia involvement was observed in 104 of 261 males and in 38 of 91 females (*P* = 0.749). Patients with and without cardia involvement also did not differ by age (*P* = 0.808).

Involvement of the fundus was uncommon (4.1% of all gastric cancer patients). It was observed in 0.7% of cases of adenocarcinoma of the cardia and in 5.6% of noncardia cases. There was no association between fundus involvement and either the sex (12/261 males and 1/91 females, *P* = 0.299) or age (*P* = 0.554) of the patients.

Metastasis was present in 60.4% of patients with adenocarcinoma of the cardia and in 59.1% of noncardia cases (*P* = 0.880). The age and sex of patients with metastasis at initial diagnosis (mean [SD], 64.2 [10.7]; 71.6% male) did not differ from those of patients without metastasis (60.9 [11.1], 72.7% male) (*P* = 0.085 for age, and *P* = 0.889 for sex). Surgery was performed for 85.7% (48) of patients without metastasis, 2.5% (2) of patients with metastasis, and 14% (6) of patients without known metastasis, but whose evaluation for metastasis was not complete.

## DISCUSSION

In many countries the incidence of esophageal cancer has increased in recent decades, while the incidence of gastric cancer has declined.^16^ However, an increasing trend in adenocarcinoma of the gastric cardia and esophagus has been reported in many European countries.^17^ This pattern has not been observed worldwide. Xiaocheng et al found that most upper gastrointestinal tract cancers among American Asian/Pacific Islanders were noncardia adenocarcinomas.^18^

The incidence of upper gastrointestinal tract cancers in Iran differs from that in Western countries—gastric cancer is the most frequent in Iran.^10^ We have previously noted a low incidence of Barrett esophagus—a precancerous lesion of the esophagus^19^—and a low incidence of esophageal adenocarcinoma^20^ in this region.

The present study reveals that noncardia gastric cancer in East Azerbaijan, Iran is more common than involvement of the cardia, which was seen in 39.2% (41.3% of adenocarcinomatous cancers) of participants. Interestingly, the ratio of gastric cancers originating from the gastric fundus is very low in this population (4.1%). Such a pattern may be due to both luminal factors and the high prevalence of *H pylori* infection.

The association between the colonization of *H pylori* in the stomach and gastric adenocarcinoma may depend on the anatomic subsite.^21^ Although currently there is no consensus,^22^ *H pylori* colonization is considered by some to be a strong risk factor for noncardia gastric cancer; however, it may be inversely associated with the risk of cancer of the gastric cardia.^23^ In 1 meta-analysis, the odds ratio for developing gastric noncardia vs cardia adenocarcinoma was 6.0, regardless of histologic subtype.^24^ The 85% prevalence of *H pylori* infection in the East Azerbaijan population^14^ probably explains the higher incidence of noncardia cancer in the present study.

A positive association has been described between intake of total, red, and processed meat and the risk of gastric noncardia cancer.^25^ This has been explained in part by a dose–response relation between red meat intake and endogenous formation of N-Nitroso compounds.^26^ There is growing evidence of an association between the Western diet (high intakes of processed meat, red meat, sweets, and high-fat dairy) and an increased risk of gastric cardia adenocarcinoma.^27^ Further comprehensive studies concerning changes in the eating habits of the Iranian population and gastric cancer patterns are required.

Regardless of the overall incidence of gastric cancer, the fraction of cardia involvement in different populations seems to follow a pattern. Gastric cardia cancer represents a low fraction (2.8%) of gastric cancers in East Asia,^28^ but an increasing trend has been reported in high-risk areas.^29^ In contrast, the rate has increased (up to 87%) in Western communities.^30^ Adenocarcinoma of the gastric cardia accounted for only 11% of gastric cancers in males and 6% in females among American Asian/Pacific islanders.^18^ In the present study, involvement of the cardia was observed in almost 40% of the patients, which is an intermediate rate. These results support our hypotheses about cardia cancers in East Azerbaijan. The relation of adenocarcinoma of the esophagus and gastroesophageal junction to gastroesophageal reflux disease is well established^31^; reports suggest an increase in the prevalence of gastroesophageal reflux disease in Iran.^32^^,^^33^

Because no published data on trends in cardia and noncardia gastric cancer among our population are available to clarify whether rates of these cancers are increasing, further investigation is required, as is the case with other high-incidence areas.

A limitation of this study was the use of a sample of patients who visited our university hospital. Although this hospital is the referral center for patients with gastrointestinal tract diseases in our area, we cannot prove that study participants were representative of gastric cancer patients in the general population of the region.

In conclusion, noncardia cancer is still the dominant subsite of gastric cancer in East Azerbaijan, which is most likely due to the very high prevalence of *H pylori* infection in the region. Additional studies of gastric cancer trends, particularly with respect to cancer subsite, would be most informative. In particular, the low ratio of cancer originating in the fundus is an obvious target for further research on the etiologic factors of gastric cancer.
